# An Alternative Device for the Topical Treatment of Oral Cancer: Development and Ex-Vivo Evaluation of Imiquimod-Loaded Polysaccharides Formulations

**DOI:** 10.3390/pharmaceutics14122573

**Published:** 2022-11-23

**Authors:** Paula de Freitas Rosa Remiro, Mariana Harue Taniguchi Nagahara, Martina Ghezzi, Alessia Filippini, Anna Demurtas, Silvia Pescina, Patrizia Santi, Cristina Padula, Ângela Maria Moraes, Sara Nicoli

**Affiliations:** 1 Department of Engineering of Materials and of Bioprocesses, School of Chemical Engineering, University of Campinas, Campinas 13083-852, Brazil; 2 ADDRes Lab, Food and Drug Department, University of Parma, Parco Area delle Scienze 27/a, 43124 Parma, Italy

**Keywords:** imiquimod, oral cancer, buccal delivery, hydrogel, alginate, xanthan gum, mucoadhesion

## Abstract

The topical use of imiquimod (IMQ), a non-specific immune response modifier, showed to be a promising therapeutic option for the early-stage treatment of some type of oral cancer, even when performed with a formulation (Aldara^®^) developed and approved for skin application. The aim of this work was the development of buccal formulations for the topical administration of IMQ with improved mucosal retention and reduced trans-mucosal permeation when compared to the reference formulation. Three different hydrogels based on carboxymethyl chitosan (CMChit), sodium alginate (A), and xanthan gum (X) in different combinations were prepared, and the loading of imiquimod was successfully performed by using a micellar formulation based on d-α-tocopheril polyethylene glycol 100 succinate (TPGS). Except for CMChit formulation, in all the other cases, the performance in vitro on the mucosa resulted comparable to the commercial formulation, despite the drug loading being 50-fold lower. Converting the gels in films did not modify the IMQ accumulated with respect to the correspondent gel formulation but produced as a positive effect a significant reduction in the amount permeated. Compared to the commercial formulation, this reduction was significant (*p* < 0.01) in the case of X film, resulting in an improvement of the retained/permeated ratio from 1 to 5.44. Mucoadhesion evaluation showed similar behavior when comparing the developed gels and the commercial formulation, and an excellent bioadhesion was observed for the films.

## 1. Introduction

Oral cancer is among the eight most common causes of cancer-related deaths [1]. Oral cancer includes cancer of the lips, tongue, cheeks, and other sites in the mouth and accounts, together with the pharynx and larynx cancers, for 10% of all cancers worldwide in men and 4% in women [2]. According to the Global Cancer Observatory, an increment of 42% in the number of new cases of oral cancer is expected in the next 20 years [3]. For the same period, the estimated number of deaths related to oral cancer is over 260 thousand, with an increment of 47% with respect to 2020 [3]. More than 90% of oral cancers are squamous cell carcinomas (SCC) [1], and currently, the first line of treatment is surgical resection [4,5]. This invasive procedure usually affects the two most important functions of the organs involved, speech and swallowing [6], which can negatively affect the patient’s quality of life. Other therapeutic options include chemotherapy, radiation therapy, and targeted therapy [5]. However, prevention and early-stage treatment of pre-cancerous lesions are preferable [7]. In this sense, the topical treatment with imiquimod seems to be a promising alternative. 

Imiquimod (IMQ) belongs to the drug class of imidazoquinolones and is a non-specific immune response modifier with potent antiviral and antitumor activity [8]. IMQ is available on the market as a 5% cream (Aldara^®^, 3M Pharmaceuticals), and although Food and Drug Administration (FDA)-approved indications include only the topical treatment of external anogenital warts, actinic keratosis and superficial basal cell carcinomas, many of the literature case-reports showed the efficacy of IMQ in the treatment of infectious, neoplastic and inflammatory skin disorders [9]. Topical IMQ off-label uses are not limited to the skin but are also reported for oral mucosa disorders. In this last case, efficacy has been proven in the topical treatment of oral dysplasia [10], human papilloma virus-related squamous cell carcinoma of the lip [11,12], mucosal melanoma [13,14], and oral squamous cell carcinoma [15]. In all the cases, the commercial cream Aldara^®^ was used. This formulation, which is intended for cutaneous use, does not have suitable organoleptic characteristics for buccal administration, and its contact time with the mucosa is limited due to the washing effect of saliva with a consequent reduction in the bioavailability and potential increase in systemic side effects. For this reason, there is an increasing request by the clinics for an IMQ formulation specially developed for buccal administration. 

The analysis of the literature shows that this topic is still little explored. To the best of our knowledge, only two formulations have been developed for this purpose. The first is a film based on polyvinylpyrrolidone (PVP), and carboxymethyl cellulose (CMC) proposed by Raminemi et al. [16,17,18], while the second was proposed by our group and is a film based on chitosan and alginate [19]. Both formulations present limitations. As the Authors state, in the case of PVP and CMC films, IMQ loading was performed by using ICH class 2 solvents such as methanol and toluene that need to be replaced, although their residual concentration in the formulation was under the limits set by the FDA guidelines. In the case of chitosan-alginate films, most of the drug is not solubilized in the matrix, and this limits its bioavailability and effective use. 

In the development of topical dosage forms for buccal application, several desirable attributes must be considered: to ensure a prolonged contact with the buccal mucosa, to guarantee the release of the loaded drug, to maximize drug retention in the tissue and, at the same time, to reduce as much as possible its systemic absorption to avoid or to reduce side effects. Among the different polymers available, we focused on polysaccharides such as chitosan derivatives, alginate, and xanthan gum. These polymers are non-toxic, water-soluble, biocompatible, and biodegradable [20,21,22], being characterized by good adhesion properties on the mucosal surface [23,24,25] and showing enhancing properties on the permeation of some drugs across the buccal mucosa [26]. 

Since the topical effect of a drug is closely related to its concentration in the target tissue, this work aimed to develop polysaccharide-based formulations that allow for high retention of imiquimod in the tissues, with limited permeation across the tissues. All developed formulations were compared to Aldara^®^, chosen as a reference. In order to incorporate the hydrophobic drug in the hydrogel, TPGS micelles were used as a solubilization strategy. Additionally, the mucoadhesive properties of the developed formulations were evaluated.

## 2. Experimental

### 2.1. Materials

Imiquimod (IMQ; IUPAC name: 1-isobutyl-1H-imidazo(4,5-c)quinoline-4-amine; MW: 240.3 g/mol; pKa: 7.3) was purchased from Hangzhou Dayangchem (Zhejiang, China). Oleic acid was purchased from Alfa Aesar (Karlsruhe, Germany), carboxymethyl chitosan (CMChit) (deacetylation degree 90%, carboxylation degree 83%) was from Santa Cruz Biotechnology (Dallas, TX, USA), sodium alginate (A) (MW 2 × 10^2^ kDa, Brookfield viscosity 2% solution in water at 25 °C 2994 cP), xanthan Gum (X) (MW 2 × 10^3^ kDa, Brookfield viscosity 1% solution in water at 25 °C 915 cP), 70% perchloric acid solution, triethylamine (TEA) and albumin from bovine serum were purchased from Sigma-Aldrich (St. Louis, MO, USA). Tocopheryl polyethylene glycol 1000 succinate (Kolliphor TPGS) was a kind gift from PMC ISOCHEM (Vert-Le-Petit, France). Carbopol 940 and sodium carboxymethyl cellulose were from ACEF (Fiorenzuola D’Arda, Italy). Mucin from the porcine stomach, Type II was from Sigma-Aldrich (Steinheim, Germany). PBS (Phosphate Buffer Saline) buffer consisted of 0.19 g/L KH_2_PO_4_, 2.37 g/L Na_2_HPO_4_, 8.8 g/L NaCl in purified water (Aerium^®^ Comfort Sartorius, Goettingen, Germany); pH 7.4 was obtained by adding 85% H_3_PO_4_. Simulated salivary fluid (SSF; 8 g/L NaCl, 2.38 g/L Na_2_HPO_4_, and 0.19 g/L KH_2_PO_4_), in purified water with pH 6.8 adjusted using 85% H_3_PO_4_, formulated according to Marques et al. [27]. For HPLC analysis, bidistilled water was used. Acetonitrile, methanol, and triethylamine were of HPLC grade; all other reagents were of analytical grade.

### 2.2. Micelles Preparation

D-α-Tocopheryl polyethylene glycol 1000 succinate (TPGS) and oleic acid micelles were prepared according to the method described by Ghezzi et al. [28]. Briefly, a micellar solution of TPGS in water (20 mM) was saturated with oleic acid (4:1, *v*/*v*). After the separation, the oily phase was removed, and the water phase was filtered twice (Minisart RC 0.2 µm, Sartorius, Gottingen, Germany). The micellar solution obtained was then saturated with IMQ by adding an excess of the drug (up to 10 mg/mL) under magnetic stirring for 3 h. Finally, the suspension was centrifuged (microcentrifuge D3024, Scilogex, Rockville, TX, USA) for 10 min at 12,500 rpm (RCF 16,600 g), and the supernatant was separated for later use. IMQ and oleic acid concentrations were 1 mg/mL and 2 mg/mL, respectively. 

### 2.3. Preparation of Gels and Films

In order to produce the gels, the polymers (xanthan gum, alginate, and CMChit in different combinations) were slowly added, at room temperature, to the micellar solution of IMQ, under magnetic stirring at 400 rpm. After 30 min, the plasticizer propylene glycol was added. In the case of formulations containing alginate, 0.5% (*w*/*v*) calcium chloride solution was added dropwise to a final content of 1% (*w*/*v*) in the formulations, and the mixtures were stirred for an additional 30 min. Gels were then deaerated by centrifugation (NEYA 16R, Remi Elektrotechnik LTD, Valiv Village, India) at 3000 rpm for 10 min. Table 1 summarizes the percent composition of the gels prepared.

For the preparation of films, 10 g of each gel formulated as described above (IMQ content 9 mg) were transferred to a glass Petri dish (5 cm in diameter) and dried at 37 °C for 24 h. The theoretical composition of the films was calculated considering the complete evaporation of the solvent after drying, and it is reported in Table 1. The film obtained from gel X was crosslinked by heat treatment in the presence of 0.03% (*w*/*v*) citric acid, according to the method described by Bueno et al. [29]. 

### 2.4. Permeation Experiments

Permeation experiments were performed using Franz-type diffusion cells with a diffusional area of 0.6 cm^2^ (DISA, Milan, Italy). The porcine esophageal epithelium was used as a model for the buccal mucosa [30,31]. The esophageal mucosa was separated from the outer muscle layer of porcine esophagi, obtained from a local slaughterhouse, with a scalpel, and the epithelium was peeled off from the connective tissue after immersion in distilled water at 60 °C for 120 s [32]. The specimens obtained were frozen and used within 3 months. The tissue, thawed at room temperature and supported by an inert membrane (regenerated cellulose, pore size 0.45 µm), was mounted between the two halves of the diffusion cell. The receptor compartment was filled with approximately 4 mL of degassed 1% *w*/*v* albumin solution in PBS pH 7.4 (IMQ solubility in this solution was 143 ± 3 µg/mL, [33]) and kept under magnetic stirring. The formulations to be tested were inserted in the donor compartment in contact with the epithelium. In the case of liquid or semisolid formulations, including the commercial cream Aldara^®^, the amount of formulations were respectively 120 µL and 400 mg (infinite dose conditions, corresponding respectively to 200 µL/cm^2^ and about 670 mg/cm^2^) while in the case of films, a circle shaped sample of 0.6 cm^2^ was used. All experiments were performed at 37 °C and lasted 6 h.

At the end of the permeation experiment, the receptor compartment was sampled, the formulations tested were removed, and the donor compartment was washed three times with 1 mL of water. After disassembling the cell, the epithelium surface was dried with filter paper and cut at the permeation area. Each tissue sample was placed in a glass vial, and the drug was extracted with the same method previously described and validated for the dermis from porcine skin [34], i.e., with 1 mL of 1:2:2 (*v*/*v*/*v*) mixture of PEG 400: methanol: HCl 1M (1:2:2, *v*/*v*/*v*) overnight at room temperature. The method resulted in being specific also in the case of the porcine esophageal epithelium (no interfering peaks with the same retention time of IMQ), and the recovery was higher than 90%.

### 2.5. HPLC Analysis

Imiquimod quantification was performed by HPLC, with a previously validated method [35] using a Flexar instrument (Perkin Elmer, Waltham, MA, USA) and a C18 column (Kinetex C18 2.6µ 100 Å 75 × 4.6 mm, Phenomenex, Torrance, CA, USA), equipped with a column guard (Widepore C18 4 × 3 mm, Phenomenex). The mobile phase was a mixture of methanol: acetonitrile: water: triethylamine (180:270:530:20, *v*/*v*/*v*/*v*) pumped at 0.5 mL/min. For samples from permeation and retention experiments, fluorescence detection was used (λ_exc_ 260 nm, λ_em_ 340 nm, injection volume: 1 µL, linearity in the range 0.05–3 µg/mL, LOD 0.01, LOQ 0.05 with RSD 2.5% and RE 16%), while for samples from release experiments, UV detection was used (λ 242 nm; injection volume: 10 µL, linearity in the range 1–10 µg/mL, LOD 0.1, LOQ 1 with RSD 4.1% and RE 2.5%). With the purpose of precipitating albumin, before HPLC analysis, the samples from the receptor compartment collected during the permeation experiments were mixed with 50 µL of 70% (*v*/*v*) perchloric acid and centrifuged for 15 min at 12,000 rpm.

### 2.6. Absorption of Artificial Saliva by the Films

The capacity of fluid absorption of the films was tested in the presence of simulated salivary fluid (SSF). Film samples of 6 mm diameter with known initial masses (*m_initial_*) were immersed in 5 mL of artificial saliva for 6 h at 37 °C and, periodically, their weights were determined (*m_humid_*) after draining the excess of the fluid with filter paper. The solution absorption capacity (*A*) was calculated using Equation (1), in grams of fluid per gram of dry film.

(1) $$A=\frac{m_{humid}-m_{initial}}{\;m_{initial}}$$

### 2.7. Imiquimod Release from the Films

The determination of drug release was performed using 6 mm in diameter disc samples of the known initial mass. All films were exposed to 5 mL of artificial saliva at 37 °C, and periodically, aliquots of 0.5 mL were collected and analyzed by HPLC to determine the imiquimod concentration. The volume of the fluid in the experiment was maintained constant by replacing the volume withdrawn with the same volume of fresh solution. The experiments lasted 8 h. 

### 2.8. Evaluation of Mucoadhesive Properties

#### 2.8.1. Rheology

Mucoadhesive properties of the developed gels were evaluated using the rheological method described by Hassan and Gallo [36] and based on the measurement of the so-called rheological synergism, represented by an increase in the polymeric solution apparent viscosity due to the interactions between polymer and mucin. A + X and X gels were prepared as described before, with the concentration of the polymers doubled and then diluted with either a mucin dispersion (6%, *w*/*w*, [37]) in SSF or neat SSF. Apparent viscosity measurements were performed with a rheometer HAAKE RheoStress 1 (ThermoFisher, Waltham, MA, USA) using a cone and plate geometry (C35/2°). The temperature was set at 37 °C, and the applied shear rate varied from 1 to 100 s^−1^. 

The viscosity component of bioadhesion (*η_b_*) was calculated using Equation (2)

(2) $$\eta_{b}=\eta_{t}-\left(\eta_{m}+\eta_{p}\right)$$

where *η_m_* is the viscosity of mucin dispersion in the absence of a polymeric solution, *η_t_* is the viscosity of mucin dispersion-polymeric solution mixture, and *η_p_* the viscosity of the polymeric solution. Since viscosity is a shear rate-dependent property, the zero-shear viscosity was calculated from the log-log plot of the viscosity curves, thus avoiding the arbitrary choice of the shear rate for the evaluation of the component of bioadhesion.

#### 2.8.2. Wash Out Test

The wash out test was adapted from Pescina et al. [38]. Briefly, approximately 15–30 mg of a semisolid formulation containing IMQ (gels and commercial formulation) or 0.6 cm^2^ film samples, accurately weighted, were applied on the surface of porcine esophageal mucosa (0.6 cm^2^) glued to a glass Petri dish (diameter 9 cm) inclined at 45°. Simulated salivary fluid (SSF) was flushed onto the formulation at a flow rate of 1 mL/min using a syringe pump (Harvard Apparatus, Inc., Holliston, MA, USA). The solution was collected at predetermined time points (every 60 s for 15 min) and analyzed by HPLC for the determination of the drug released. Experiments were performed at room temperature (19–22 °C). For the preparation of the tissue, the esophageal mucosa was separated from the outer muscle layer with a scalpel, cut in circles of 0.6 cm^2,^ and frozen until use. 

### 2.9. Statistical Analysis

Data were reported as mean value ± sd. The significance of differences between values were assessed using one-way ANOVA, followed by Dunnet’s test or Tukey’s test, and *t*-test (Prism, GraphPad Software, San Diego, CA, USA). Differences were considered statistically significant when *p* < 0.05. 

## 3. Results and Discussion

Considering the demonstrated efficacy of Aldara^®^ cream in the treatment of oral cavity lesions and the fact that this formulation is not adequate for use in this region, in this work, we propose a formulation specifically designed for IMQ buccal administration. With this formulation, we intend to provide prolonged drug contact with the buccal mucosa, maximizing IMQ retention in the tissue and, at the same time, reducing as much as possible its systemic absorption.

### 3.1. Preliminary Experiments

The first step of this work concerned the evaluation of the retention and permeation of IMQ in ex-vivo experiments starting from the commercial formulation Aldara^®^ to define the target therapeutically effective concentration in the tissue. Porcine esophageal epithelium, a reasonable and well-characterized model for the human buccal mucosa [30,39], was used as a barrier in the ex-vivo studies. Aldara^®^ was applied in infinite dose conditions (about 670 mg/cm^2^, corresponding to 33.5 mg/cm^2^ of IMQ), and the obtained amount of IMQ retained in the tissue and permeated across the tissue are given in Table 2. 

For the commercial cream, the amount of IMQ accumulated in and across the porcine tissue was very similar, with a retained/permeated ratio of about 1. 

By aiming at ensuring prolonged contact time with the mucosa, a possible formulation could be a gel based on mucoadhesive polymers. The polymers initially evaluated for the preparation of the gel were CMChit, xanthan gum, and sodium alginate. These polysaccharides were selected on the basis of their mucoadhesive characteristics and on the basis of our previous experience in combining IMQ with alginate and chitosan [19]. Since IMQ is poorly soluble in aqueous media, drug addition to the gel was performed either as a suspension (IMQ micronized powder, obtained as described in [40] dispersed in the gel) or as an emulsion (IMQ solubilized in isostearic acid, that was subsequently emulsified with the aqueous gel). The gel composition is reported in the supplementary material (Table S1). Different drug concentrations and polymer combinations were evaluated. Nevertheless, no IMQ was recovered in the tissue nor in the receptor compartment. This result suggests that a solubilizing and enhancing strategy is needed. Probably, the relatively low amount of isostearic acid that can be loaded in the gel is not enough to solubilize a relevant amount of IMQ and promote its penetration. 

### 3.2. Polymeric Micelles and Micelles-Based Gels

**Figure 1:**
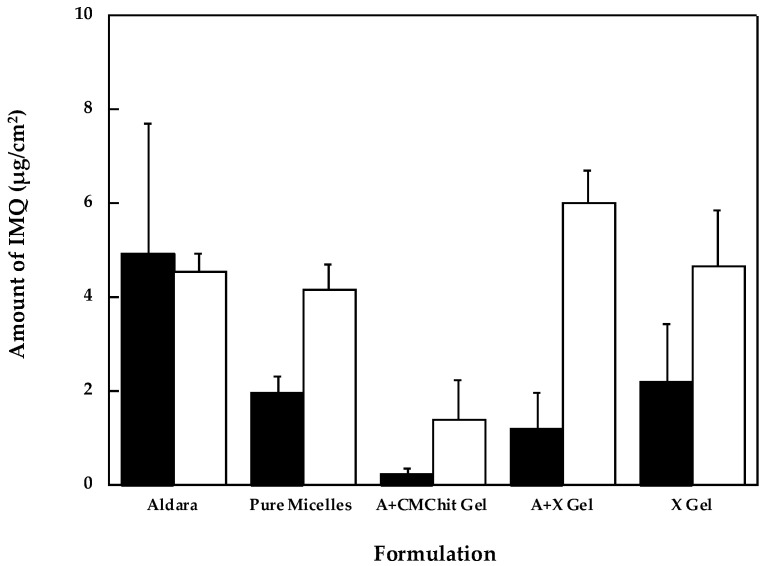
IMQ retained (dark bar) and permeated (light bar) across porcine esophageal epithelium from gels (mean ± sd).

A different strategy was then adopted. In a previous work, TPGS-based micelles co-loaded with oleic acid were successfully developed to improve IMQ solubility and effectively promote drug retention inside the skin [28]. These micelles are characterized by a lipophilic core made of vitamin E and a hydrophilic PEG corona; the presence of oleic acid (2 mg/mL) is necessary to improve IMQ loading that resulted in being 1 mg/mL. Micelles are approximately 13 nm in diameter, and their zeta potential is close to zero [28]. Initially, this micellar solution was tested as it was, applied to the esophageal mucosa in infinite dose conditions (200 µL/cm^2^). Compared to Aldara^®^ (Figure 1), the micellar solution produced a lower mucosal level, even if not significantly different, and a similar permeated drug amount. 

However, the most encouraging aspect of this result is that, despite the IMQ concentration in the micellar solution being 50 times lower than in the commercial cream (0.1 vs. 5% respectively), the performance on the mucosa is comparable, confirming the permeation enhancer ability of these polymeric micelles also on the mucosa. Even though the wide use of TPGS for drug delivery [41] and the demonstration of its permeation-enhancing activity on the skin [42,43,44] and on corneal [44,45,46], conjunctival [47,48] and nasal mucosa [49], very few data are found in the literature on its buccal application. Among these, to the best of our knowledge, only two works report the effect of TPGS micelles on the permeation of drugs across the buccal mucosa. In particular, Basahih et al. [50] successfully improved glimepiride permeation across goat buccal mucosa by encapsulating it in TPGS micelles incorporated in HPMC (hydroxypropyl methylcellulose) and carbomer film, while Suksiriworapong et al. [51] showed that mucoadhesion and itraconazole permeation across the porcine buccal mucosa can be improved by the presence of thiolic groups on TPGS micelles surface.

Since the micellar formulation is a liquid, it is not suitable for the prolonged application, but it can be used as the base for the preparation of gels. In particular, based on previous experience with polymeric mixtures [52], gel consisting of a combination of CMChit and alginate, of xanthan gum and alginate and xanthan gum alone were evaluated. The composition of the three gels is detailed in Table 1, and the obtained results are presented in Figure 1 (the complete statistical analysis of the differences, evaluated with a *t*-test, is reported in supplementary material, Table S2). Thickening of micellar solution with CMChit brought a significant reduction in mucosal permeation and retention (*p* < 0.01, *t*-test), while the use of xanthan gum and alginate (A + X) or xanthan gum alone (X) gave rise to comparable results with respect to un-thickened micelles with the exception of significant (*p* < 0.005) increase in amount permeated in the case of A + X gel. Differences among the gels can be due to the different viscosity of the three vehicles, together with a different capacity of oleic acid/TPGS micelles to diffuse across the gel to reach the mucosa surface. Indeed, previous data have highlighted the capability of these micelles to diffuse across negatively charged natural polymers, such as xanthan gum, while, in other cases, micelles remained trapped in the polymeric network [28].

### 3.3. Films

Despite the use of mucoadhesive polymers being attractive, the use of semisolid formulations does not fully meet the requirement of maintaining the formulation in place for long periods. Additionally, the low retained/permeated ratio (see Table 2) showed that these formulations might not be suitable for local delivery. Films can represent a relevant alternative since, if showing mucoadhesive properties, they can stick to the mucosal lesion, release the drug in a controlled manner and protect the lesion from contaminants and mild mechanical stimuli. Furthermore, it is possible to add an impermeable backing layer to avoid drug release into the oral cavity and reduce systemic absorption while also further increasing the protective function.

For this reason, the formulated gels were processed into films by the classical casting approach. In the case of A + CMChit formulation, the obtained film was not homogeneous, and for this reason, it was not further investigated. 

**Figure 2:**
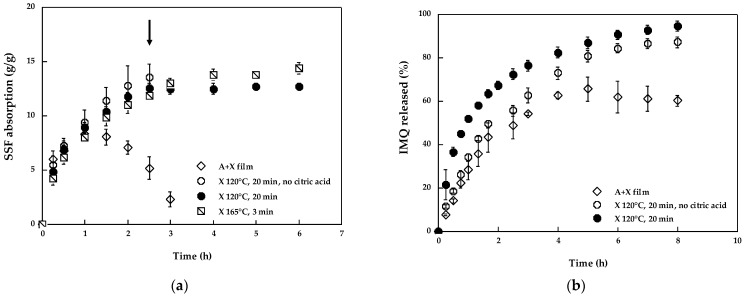
(**a**) Swelling profile of films in SSF (mean ± sd). The arrow indicates the loss of cohesion of the hydrogel; (**b**) IMQ release from films (mean ± sd).

The composition of the two films based on alginate and/or xanthan gum is detailed in Table 1. Given the presence of the micelles in the formulation, the preponderant percentage of the film is represented by TPGS (between 51 and 61%). Alginate and xanthan gum, despite being added in a lower percentage, have mucoadhesive and film-forming properties, while film flexibility was achieved by the addition of propylene glycol as the plasticizer. The characteristics of the films in terms of thickness and weight per unit area are also reported in Table 1, while the results of swelling studies and drug release in SSF are reported in Figure 2.

The film containing both alginate and xanthan gum (A + X film) was crosslinked by the presence of CaCl_2_. Ca^2+^ coordinates the l-guluronic acid residues of different polymer chains, allowing the formation of a three-dimensional network described in the literature as the egg-box model [53] probably intertwined with xanthan gum chains. Its swelling behavior was evaluated initially in distilled water, and these preliminary data showed that the maximum uptake is reached after 4 h, and it maintains its integrity for a period of at least 24 h, suitable for buccal application. Instead, for this work, simulated saliva (SSF) [54,55,56] was used to analyze the performance of the biomaterials in a micro-environment more closely related to the physiological conditions of the mouth. Contrary to what was observed in the swelling experiments in water, in the case of the SSF (Figure 2a), the maximum uptake was observed after 1 h, and gradual erosion of the film was observed within two hours with simultaneous formation of an insoluble precipitate. This is probably due to the presence of phosphate ions in the solution, which, by sequestering calcium ions, lead to a destabilization of the structure and its fragmentation. IMQ release (Figure 2b) in these conditions is not complete, and the apparent reduction in the released percentage after 5 h suggests an interference of the calcium phosphate precipitation with IMQ solubility.

The film based on xanthan gum (X film) rapidly dissolved in contact with SSF. To stabilize it, citric acid, a natural and non-toxic compound, was added, and the film was crosslinked by heat treatment according to the protocol of Bueno et al. [29]. The first attempt was carried out at 165 °C for 7 min, as described in the literature. In these conditions, however, pseudo-carbonization of the film occurred, and the duration of the treatment was then reduced to 3 min. The film obtained showed a relatively fast SSF uptake (Figure 2a) and maintained its integrity for at least 6 h. Since high temperature can represent a problem for the stability of drugs and TPGS, and since the condensation process (ester links) is both temperature and time-dependent, less drastic conditions were evaluated, namely 140 °C for 30 min, 130 °C for 25 min and 120 °C for 20 min. The exposure of the film to the lowest temperature for the shortest heating time (120 °C for 20 min) was enough to obtain a similar absorption profile and stability, suggesting a comparable crosslinking degree. The cross-linking of xanthan gum in the presence of citric acid and heat treatment is the consequence of the formation of ester links between the polysaccharide chains and citric acid following a condensation reaction [29]. In the absence of citric acid, however, the condensation process occurs equally with the formation of intra- and intermolecular ester bonds, but in this case, the crosslinking density is reduced because of the lower availability of groups that can be crosslinked [29]. The effect of the lower crosslinking density on the swelling of the film is reported in Figure 2a (A 120 °C 20 min no citric acid), and the results obtained showed an initial swelling followed by the gradual transformation of the film into a gel within 2 to 3 h, supporting the role of crosslinking density on the stability and dissolution of the film. Film conversion to a gel reflects in a less regular IMQ release profile (Figure 2b). 

The film crosslinked at 120 °C for 20 min in the presence of citric acid gradually releases the drug up to 8 h when almost the entire content has been released. Interestingly, until the complete film swelling (2.5 h), only 65% of the drug has been released. The swollen matrix-controlled drug release in the following 6 h. Given the very low aqueous solubility of IMQ and the demonstrated capability of TPGS micelles to diffuse into X gels [28], it is reasonable to hypothesize that drug-loaded micelles are released from the gel.

#### Drug Retention and Permeation

**Figure 3:**
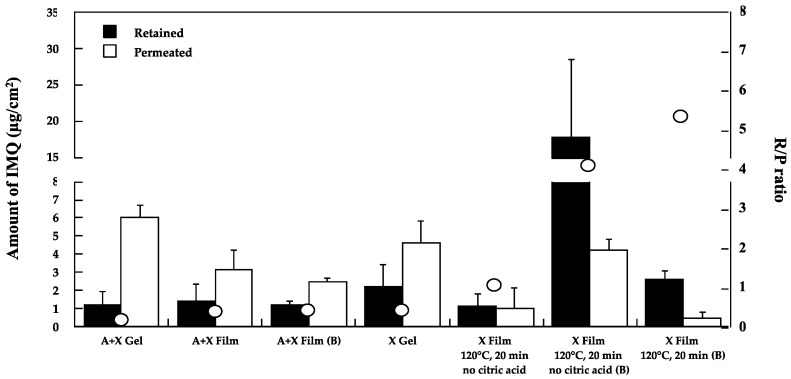
IMQ retained (dark bar) and permeated (light bar) across porcine esophageal epithelium from A + X and X formulations (mean ± sd); circles indicate R/P ratio.

The performance of the prepared A + X and X films (crosslinked and not crosslinked) was then evaluated on the mucosa, and the results are reported in Figure 3. 

The transformation of the A + X gel into a crosslinked film did not modify the amount of IMQ accumulated but produced, as a positive effect, a significant reduction (*p* < 0.05) of the amount permeated with a slight increase in the R/P ratio from 0.20 to 0.44. In order to guarantee the unidirectionality of the release, the A + X film was also tested in the presence of an impermeable backing layer (A + X Film (B)). The presence of the backing did not substantially modify the performance of the film. This result was notable because, in view of in-vivo administration, it represents a valuable strategy to further reduce systemic absorption. 

When the X gel was transformed into a film, crosslinked by heat treatment only (120 °C, 20 min, no citric acid), a similar behavior was noted: the amount retained is comparable with that of the gel, while a statistically significant reduction in the amount permeated was observed (*p* < 0.05) (see Figure 3). Differently from the A + X film, the application of an impermeable backing layer, which allowed for the maintenance of the water content of the film, increased the IMQ retention and permeation even if, due to the great variability of the data, the increase was significant only in the case of the amount permeated (*p* < 0.05). The chemical crosslinking of X film with citric acid lowered the variability of the data and, in the same application conditions (i.e., with backing layer), produced a marked reduction (*p* < 0.001) of IMQ permeated. The performance of this film was particularly interesting: compared to the commercial formulation, the amount retained is approximately the same, and, above all, a 10-fold reduction in the amount permeated was observed. This result, together with the lower amount applied and the unidirectionality of the release (provided by the use of the impermeable backing), could substantially reduce the risk of systemic absorption. 

### 3.4. Mucoadhesion Studies

The evaluation of mucoadhesion is an essential step for the development of a buccal drug delivery system, and many in-vitro methods are available, including, among others, rheology, tensile tester, atomic force microscopy, ellipsometry, and flow channel analysis [57]. Mucoadhesion properties, defined as the ability of a material to adhere to a mucosal surface, are essential to produce prolonged contact with the absorption site and, consequently, to improve drug absorption. Although the mechanisms are not yet entirely understood, in the mucoadhesion phenomenon, three phases have been identified: (i) the contact between the hydrated polymer and the mucosa, (ii) the interpenetration of the polymer chains and mucin, the main macromolecular component of mucus, and (iii) the formation of bonds between the polymer and mucin chains [58]. For the evaluation of the mucoadhesive properties of the developed hydrogels, the rheological analysis approach was used. This method, proposed first by Hassan and Gallo [36], is based on the evaluation of the rheological synergism that is a more than the additive increase in viscosity that occurs when mucoadhesive polymer solution and mucin dispersion are mixed. Since for gels drug loading, IMQ was solubilized in TPGS-based micelles, to evaluate the effect of the latter on mucoadhesion, gels were prepared both in water and TPGS micellar solution. Two well-known mucoadhesive polymers such as Carbopol 940 and sodium carboxymethylcellulose (NaCMC), were analyzed as well to serve as comparative standards.

As can be observed from the values of the viscosity component of bioadhesion (*η_b_*) reported in Table 3, both hydrogels exhibited a positive rheological synergism (*η_b_* > 0) when mixed with the mucin dispersion in the same order of magnitude of the polymers used as references. This positive behavior has already been observed for xanthan gum and sodium alginate separately [59,60].

In the conditions used, i.e., pH value near neutrality, both polymers and mucin are negatively charged [59,61]. The repulsion arising from the ionized functional groups induces the uncoiling of the polymer chains, which, in turn, facilitates chain entanglement and secondary interaction [59], leading to a consequent increase in viscosity. The presence of alginate in the A + X formulation increases significantly (*p* < 0.05) the viscosity component of bioadhesion, probably thanks to an additive effect and to the raised total polymer content of the formulation. Moreover, the addition of Ca^2+^ ions for alginate cross-linking, is known to increase the interaction between polymer and mucin [60]. For both gels, the presence of TPGS micellar solution does not significantly affect the mucoadhesion. The viscosity component of bioadhesion was also evaluated for the neat micellar solution. The negative value obtained (−0.20 ± 0.02) indicates poor bioadhesion, probably due to the neutral charge of the micelles, as already demonstrated by Suksiriworapong et al. [51]. 

With the aim of assessing mucoadhesion in a more in-vivo-like condition, the washout test was also performed. This method, also suitable for the evaluation of solid formulations such as films, is often employed to measure mucoadhesion of dosage forms intended to be administered in regions of the human body where the mucosal tissues are subject to continuous fluid flow, as in the case of the oral cavity. This method involves a dynamic setup that simulates the biological flow that washes the dosage form from the mucosal surface. The amount of drug remaining on the mucosal surface can be determined by analyzing the content of the drug in the collected perfusate. Thus, with this method, two different phenomena contribute to the results: mucoadhesion and drug release from the formulation. 

**Figure 4:**
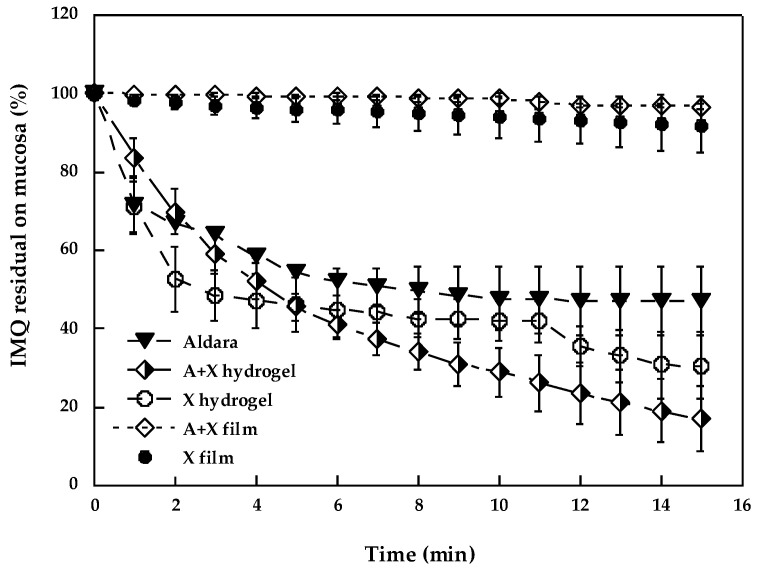
IMQ residual on mucosa during the washout test (mean ± sd).

Figure 4 reports the percentage of IMQ present on the mucosa as a function of time for the different formulations prepared. For comparison purposes, Aldara^®^ was also analyzed. 

The commercial formulation, although not designed for the buccal application, showed to be slightly mucoadhesive: after 15 min, about 50% of the applied dose was still present on the mucosa. Both the developed hydrogels exhibited similar behavior, which resulted in being significantly lower (*p* < 0.05) compared to Aldara^®^ only for the A + X gel in the interval of 8–15 min. The relatively good performance of Aldara^®^ in comparison with the gels can be attributed to a slower drug release. In fact, while in the case of the gels, IMQ is loaded using hydrophilic TPGS micelles, having high affinity for the simulated salivary fluid, in the case of Aldara^®^ (an O/W emulsion), the drug is dispersed into isostearic acid droplets which, being lipophilic are cleared more slowly by the SSF. 

The behavior of the films with respect to the semisolid formulations is different, even if similar to each other. Both exhibit a residual amount of IMQ of approx. 90% after 15 min, significantly higher (*p* < 0.01) than those obtained with all the tested semisolid formulations for the whole test period, suggesting an excellent bioadhesion and a controlled release of the loaded drug. 

Limited to the gel formulations, the correlation between the mucoadhesion results obtained with the two approaches is poor, mostly because of the different set-ups used; while the rheology study is focused on polymer-mucin interaction and gives an only indication of the possible persistence of the formulation in contact with the tissue, the inclined plane set-up combines mucoadhesion with drug release, providing complementary information. The association of the two methods can be useful for polymer selection, elucidate the mechanisms involved in mucoadhesion, and evaluate the release of the drug in more in vivo-like conditions.

## 4. Conclusions

Due to its well-known solubility limitations, IMQ delivery system formulation is particularly challenging, especially in the case of water-based formulations. The use of IMQ encapsulated in TPGS and oleic acid micelles allowed to successful load of IMQ in aqueous polysaccharide-based formulations and enhanced its penetration and retention in the mucosal tissue. Indeed, both the type of formulations, gels and films, produced IMQ tissue levels similar to that of the commercial cream Aldara^®^ used as a reference, despite a 50-fold lower IMQ concentration, indicating a higher efficiency of the developed formulations. The best performing formulation, a xanthan gum crosslinked film that produced IMQ concentration in the tissue similar to the commercial formulation but with a marked reduction in the amount of IMQ permeated across the tissue, could probably likely result in a safer formulation. Finally, the film displayed excellent mucoadhesion properties and a controlled release of the drug. Taken together, the results obtained in the present work are encouraging and could represent a good starting point for providing clinicians with the needed IMQ buccal formulation.

## Figures and Tables

**Table 1:** Percent composition (*w*/*w*) of the formulations tested.

Component	TPGS Micellar Solution	A + CMChit Gel	A + X Gel	X Gel	A + X Film ^(a)^	X Film ^(a)^
IMQ	0.10	0.09	0.09	0.08	1.71	1.70
TPGS	3.00	2.65	2.67	2.95	51.28	61.06
Oleic acid	0.20	0.21	0.18	0.20	3.42	4.07
Water	96.70	94.18	94.80	95.17	-	-
Alginate	-	0.88	0.89	-	17.01	-
Xanthan gum	-	-	0.44	0.59	8.55	12.21
Propylene glycol	-	1.06	0.89	0.98	17.09	20.35
Carboxymethyl chitosan	-	0.88	-	-		-
Calcium chloride	-	0.04	0.04	-	0.85	-
Citric acid	-	-	-	-	-	0.61
***Thickness (mm)***					*0.36 ± 0.01*	*0.26 ± 0.01*
***Weight (mg*/*cm^2^)***					*35.62 ± 1.36*	*27.44 ± 1.28*

^(a)^ theoretical composition considering the complete solvent evaporation.

**Table 2:** IMQ retained and permeated across porcine esophageal epithelium from topical formulations (mean ± sd).

Formulation	IMQ Retained (µg/cm^2^)	IMQ Permeated (µg/cm^2^)	IMQ Retained and Permeated Ratio (R/P)
Aldara^®^	4.91 ± 2.79	4.55 ± 0.39	1.08
TPGS micellar solution	1.98 ± 0.33	4.16 ± 0.54	0.48
A + CMChit gel	0.22 ± 0.14	1.4 ± 0.83 ^(c)^	0.16
A + X gel	1.21 ± 0.74	6.01 ± 0.67	0.20
X gel	2.18 ± 1.23	4.65 ± 1.18	0.47
A + X Film	1.38 ± 0.99	3.16 ± 1.08	0.44
A + X Film with backing	1.18 ± 0.23	1.98 ± 1.84 ^(a)^	0.60
X Film not crosslinked	1.14 ± 0.68	1.04 ± 1.11 ^(c)^	1.10
X Film not crosslinked with backing	17.76 ± 10.70 ^(b)^	4.24 ± 0.57	4.19
X Film 120 °C 20 min with backing	2.61 ± 0.46	0.48 ± 0.36 ^(c)^	5.44

The significance level of differences was evaluated with one-way ANOVA, followed by Dunnet’s test considering Aldara^®^ formulation as the control: ^(a)^ *p* < 0.05; ^(b)^ *p* < 0.01; ^(c)^ *p* < 0.001.

**Table 3:** Polymer solution viscosity (*η_p_*) and viscosity component of bioadhesion (*η_b_*) (mean value ± sd).

Polymer	Solvent	*η_p_* (Pa·s)	*η_b_* (Pa·s)	Significance Level of Differences
A + X	Water	9.81 ± 1.28	4.76 ± 1.05	*p* < 0.05 vs. X in water
A + X	TPGS micellar solution	12.33 ± 0.91	6.38 ± 2.11	*p* < 0.01 vs. X in water and X micellar
X	Water	3.18 ± 0.56	1.40 ± 0.26	n.s.
X	TPGS micellar solution	1.95 ± 0.61	1.81 ± 0.15	n.s.
*Carbopol 940 (1%)*	*Water*	*12.11* ± 0.12	*1.53* ± 0.55	*n.d.*
*NaCMC (4%)*	*Water*	*7.56* ± 1.13	*3.35 ± 0.41*	*n.d.*

n.s.: non significance. n.d.: non difference.

## Data Availability

Data are available upon justified request.

## Supplementary Materials

The following supporting information can be downloaded at: https://www.mdpi.com/article/10.3390/pharmaceutics14122573/s1, Table S1: Percentage composition of the preliminary formulations.; Table S2: P values obtained from *t*-test analysis for IMQ amount retained and permeated across porcine skin for all the formulations prepared.

## References

[1] Johnson N.W., Jayasekara P., Amarasinghe A.A. (2011). Squamous cell carcinoma and precursor lesions of the oral cavity: Epidemiology and aetiology. Periodontology 2000.

[2] https://www.airc.it/cancro/informazioni-tumori/guida-ai-tumori/tumore-della-bocca#:~:text=L'incidenza%20in%20Italia%20%C3%A8,3%20casi%20ogni%20100.000%20femmine.

[3] https://gco.iarc.fr/.

[4] Montero P.H., Patel S.G. (2015). Cancer of the oral cavity. Surg. Oncol. Clin. N. Am..

[5] Machiels J.P., Leemans C.R., Golusinski W., Grau C., Licitra L., Gregoire V. (2020). Squamous cell carcinoma of the oral cavity, larynx, oropharynx and hypopharynx: EHNS-ESMO-ESTRO Clinical Practice Guidelines for diagnosis, treatment and follow-up. Ann. Oncol..

[6] Kolokythas A. (2010). Long-term surgical complications in the oral cancer patient: A comprehensive review. Part I. J. Oral Maxillofac. Res..

[7] Sankaranarayanan R., Ramadas K., Amarasinghe H., Subramanian S., Johnson N., Gelband H., Jha P., Sankaranarayanan R., Horton S. (2015). Oral Cancer: Prevention, Early Detection, and Treatment. Cancer: Disease Control Priorities.

[8] Sauder D.N. (2000). Immunomodulatory and pharmacologic properties of imiquimod. J. Am. Acad. Dermatol..

[9] Hanna E., Abadi R., Abbas O. (2016). Imiquimod in dermatology: An overview. Int. J. Dermatol..

[10] Mullins R., Ansell M., Laverick S. (2016). Treatment of oral dysplasia with 5% imiquimod cream: Short communication. Br. J. Oral Maxillofac. Surg..

[11] Esquivel-Pedraza L., Fernandez-Cuevas L., Saeb-Lima M., Guerrero-Ramos B.A., Hernandez-Salazar A., Mendez-Flores S. (2015). Recalcitrant oral squamous cell papilloma lesions in two HIV-infected patients successfully treated with topical imiquimod. J. Dermatol. Case Rep..

[12] Pentangelo G., Nistico S.P., Provenzano E., Cisale G.Y., Bennardo L. (2021). Topical 5% Imiquimod Sequential to Surgery for HPV-Related Squamous Cell Carcinoma of the Lip. Medicina.

[13] Satish T., Khan S., Levin M., Carvajal R., Yoon A.J. (2021). Treatment of recurrent mucosal melanoma of the oral cavity with topical imiquimod and pembrolizumab achieves complete histopathologic remission. J. Immunother. Cancer.

[14] Spieth K., Kovacs A., Wolter M., Bug R., Kaufmann R., Gille J. (2006). Topical imiquimod: Effectiveness in intraepithelial melanoma of oral mucosa. Lancet Oncol..

[15] Wester A., Eyler J.T., Swan J.W. (2017). Topical imiquimod for the palliative treatment of recurrent oral squamous cell carcinoma. JAAD Case Rep..

[16] Ramineni S.K., Cunningham L.L., Dziubla T.D., Puleo D.A. (2013). Development of imiquimod-loaded mucoadhesive films for oral dysplasia. J. Pharm. Sci..

[17] Ramineni S.K., Cunningham L.L., Dziubla T.D., Puleo D.A. (2013). Competing Properties of Mucoadhesive Films Designed for Localized Delivery of Imiquimod. Biomater. Sci..

[18] Ramineni S.K., Dziubla T.D., Cunningham L.L., Puleo D.A. (2014). Local delivery of imiquimod in hamsters using mucoadhesive films and their residence time in human patients. Oral Surg. Oral Med. Oral Pathol. Oral Radiol..

[19] Camargo L.G., de Freitas Rosa Remiro P., Rende G.S., di Carla Santos S., Fraz-Montan M., Moraes A.M. (2021). Development of bioadhesive polysaccharide-based films for topical release of the immunomodulatory agent imiquimod on oral mucosa lesions. Eur. Polym. J..

[20] Cortes H., Caballero-Floran I.H., Mendoza-Munoz N., Escutia-Guadarrama L., Figueroa-Gonzalez G., Reyes-Hernandez O.D., Gonzalez-Del Carmen M., Varela-Cardoso M., Gonzalez-Torres M., Floran B. (2020). Xanthan gum in drug release. Cell. Mol. Biol..

[21] Shariatinia Z. (2018). Carboxymethyl chitosan: Properties and biomedical applications. Int. J. Biol. Macromol..

[22] Hariyadi D.M., Islam N. (2020). Current Status of Alginate in Drug Delivery. Adv. Pharmacol. Pharm. Sci..

[23] Ways T.M., Lau W.M., Khutoryanskiy V. (2018). Chitosan and Its Derivatives for Application in Mucoadhesive Drug Delivery Systems. Polymers.

[24] Lehr C.M., Bouwstra J.A., Schacht E.H., Junginger H.E. (1992). In vitro evaluation of mucoadhesive properties of chitosan and some other natural polymers. Int. J. Pharm..

[25] Zeng N., Seguin J., Destruel P.L., Dumortier G., Maury M., Dhotel H., Bessodes M., Scherman D., Mignet N., Boudy V. (2017). Cyanine derivative as a suitable marker for thermosensitive in situ gelling delivery systems: In vitro and in vivo validation of a sustained buccal drug delivery. Int. J. Pharm..

[26] Sandri G., Poggi P., Bonferoni M.C., Rossi S., Ferrari F., Caramella C. (2006). Histological evaluation of buccal penetration enhancement properties of chitosan and trimethyl chitosan. J. Pharm. Pharmacol..

[27] Marques M.R.C., Loebenberg R., Almukainzi M. (2011). Simulated biological fluids with possible application in dissolution testing. Dissolution Technol..

[28] Ghezzi M., Pescina S., Delledonne A., Ferraboschi I., Sissa C., Terenziani F., Remiro P.F.R., Santi P., Nicoli S. (2021). Improvement of Imiquimod Solubilization and Skin Retention via TPGS Micelles: Exploiting the Co-Solubilizing Effect of Oleic Acid. Pharmaceutics.

[29] Bueno V.B., Bentini R., Catalani L.H., Petri D.F. (2013). Synthesis and swelling behavior of xanthan-based hydrogels. Carbohydr. Polym..

[30] Del Consuelo I.D., Pizzolato G.P., Falson F., Guy R.H., Jacques Y. (2005). Evaluation of pig esophageal mucosa as a permeability barrier model for buccal tissue. J. Pharm. Sci..

[31] Diaz-Del Consuelo I., Jacques Y., Pizzolato G.P., Guy R.H., Falson F. (2005). Comparison of the lipid composition of porcine buccal and esophageal permeability barriers. Arch. Oral Biol..

[32] Padula C., Pozzetti L., Traversone V., Nicoli S., Santi P. (2013). In vitro evaluation of mucoadhesive films for gingival administration of lidocaine. AAPS PharmSciTech.

[33] Telo I., Favero E.D., Cantu L., Frattini N., Pescina S., Padula C., Santi P., Sonvico F., Nicoli S. (2017). Gel-like TPGS-Based Microemulsions for Imiquimod Dermal Delivery: Role of Mesostructure on the Uptake and Distribution into the Skin. Mol. Pharm..

[34] Telo I., Pescina S., Padula C., Santi P., Nicoli S. (2016). Mechanisms of imiquimod skin penetration. Int. J. Pharm..

[35] Pescina S., Garrastazu G., Del Favero E., Rondelli V., Cantu L., Padula C., Santi P., Nicoli S. (2018). Microemulsions based on TPGS and isostearic acid for imiquimod formulation and skin delivery. Eur. J. Pharm. Sci..

[36] Hassan E.E., Gallo J.M. (1990). A simple rheological method for the in vitro assessment of mucin-polymer bioadhesive bond strength. Pharm. Res..

[37] Ivarsson D., Wahlgren M. (2012). Comparison of in vitro methods of measuring mucoadhesion: Ellipsometry, tensile strength and rheological measurements. Colloids Surf. B Biointerfaces.

[38] Pescina S., Macaluso C., Gioia G.A., Padula C., Santi P., Nicoli S. (2017). Mydriatics release from solid and semi-solid ophthalmic formulations using different in vitro methods. Drug Dev. Ind. Pharm..

[39] Telo I., Tratta E., Guasconi B., Nicoli S., Pescina S., Govoni P., Santi P., Padula C. (2016). In-vitro characterization of buccal iontophoresis: The case of sumatriptan succinate. Int. J. Pharm..

[40] de Freitas Rosa Remiro P., de Tarso Vieira e Rosa P., Moraes A.M. (2022). Effect of process variables on imiquimod micronization using a supercritical antisolvent (SAS) precipitation technique. J. Supercrit. Fluid..

[41] Luiz T.M., Di Filippo D.M., Alves R.C., Araùjo S.V.H., Duarte L.J., Marchetti M.J., Chorilli M. (2021). The use of TPGS in drug delivery systems to overcome biological barriers. Eur. Polym. J..

[42] Ghosh I., Michniak-Kohn B. (2012). Design and characterization of submicron formulation for a poorly soluble drug: The effect of Vitamin E TPGS and other solubilizers on skin permeability enhancement. Int. J. Pharm..

[43] Romero G.B., Arntjen A., Keck C.M., Muller R.H. (2016). Amorphous cyclosporin A nanoparticles for enhanced dermal bioavailability. Int. J. Pharm..

[44] Ghezzi M., Ferraboschi I., Delledonne A., Pescina S., Padula C., Santi P., Sissa C., Terenziani F., Nicoli S. (2022). Cyclosporine-loaded micelles for ocular delivery: Investigating the penetration mechanisms. J. Control. Release.

[45] Caruso C., Porta A., Tosco A., Eletto D., Pacente L., Bartollino S., Costagliola C. (2020). A Novel Vitamin E TPGS-Based Formulation Enhances Chlorhexidine Bioavailability in Corneal Layers. Pharmaceutics.

[46] Ostacolo C., Caruso C., Tronino D., Troisi S., Laneri S., Pacente L., Del Prete A., Sacchi A. (2013). Enhancement of corneal permeation of riboflavin-5′-phosphate through vitamin E TPGS: A promising approach in corneal trans-epithelial cross linking treatment. Int. J. Pharm..

[47] Pescina S., Lucca L.G., Govoni P., Padula C., Favero E.D., Cantu L., Santi P., Nicoli S. (2019). Ex Vivo Conjunctival Retention and Transconjunctival Transport of Poorly Soluble Drugs Using Polymeric Micelles. Pharmaceutics.

[48] Somavarapu S., Pandit S., Gradassi G., Bandera M., Ravichandran E., Alpar O.H. (2005). Effect of vitamin E TPGS on immune response to nasally delivered diphtheria toxoid loaded poly(caprolactone) microparticles. Int. J. Pharm..

[49] Ahmed T.A., El-Say K.M., Ahmed O.A., Aljaeid B.M. (2019). Superiority of TPGS-loaded micelles in the brain delivery of vinpocetine via administration of thermosensitive intranasal gel. Int. J. Nanomed..

[50] Basahih T.S., Alamoudi A.A., El-Say K.M., Alhakamy N.A., Ahmed O.A.A. (2020). Improved Transmucosal Delivery of Glimepiride via Unidirectional Release Buccal Film Loaded with Vitamin E TPGS-Based Nanocarrier. Dose Response.

[51] Suksiriworapong J., Mingkwan T., Chantasart D. (2017). Enhanced transmucosal delivery of itraconazole by thiolated d-a-tocopheryl poly(ethylene glycol) 1000 succinate micelles for the treatment of Candida albicans. Eur. J. Pharm. Biopharm..

[52] Westin C.B., Nagahara M.H.T., Decarli M.C., Kelly D.J., Moraes A.M. (2020). Development and characterization of carbohydrate-based thermosensitive hydrogels for cartilage tissue engineering. Eur. Polym. J..

[53] Cao L., Lu W., Mata A., Nishinari K., Fang Y. (2020). Egg-box model-based gelation of alginate and pectin: A review. Carbohydr. Polym..

[54] Gohel M.C., Parikh R.K., Aghara P.Y., Nagori S.A., Delvadia R.R., Dabhi M.R. (2009). Application of simplex lattice design and desirability function for the formulation development of mouth dissolving film of salbutamol sulphate. Curr. Drug Deliv..

[55] de Almeida Pdel V., Gregio A.M., Machado M.A., de Lima A.A., Azevedo L.R. (2008). Saliva composition and functions: A comprehensive review. J. Contemp. Dent. Pract..

[56] Humphrey S.P., Williamson R.T. (2001). A review of saliva: Normal composition, flow, and function. J. Prosthet. Dent..

[57] Woertz C., Preis M., Breitkreutz J., Kleinebudde P. (2013). Assessment of test methods evaluating mucoadhesive polymers and dosage forms: An overview. Eur. J. Pharm. Biopharm..

[58] Jiménez-Castellano R., Zia H., Rhodes C.T. (1993). Mucoadhesive drug delivery systems. Drug Dev. Ind. Pharm..

[59] Ahmad M., Ritzoulis C., Chen J., Meigui H., Bushra R., Jin Y., Xiao H. (2021). Xanthan gum-mucin complexation: Molecular interaction, thermodynamics, and rehological analysis. Food Hydrocoll..

[60] Fuongfuchat A., Jamieson A.M., Blackwell J., Gerken T.A. (1996). Rheological studies of the interaction of mucins with alginate and polyacrylate. Carbohydr. Res..

[61] Khutoryanskiy V.V. (2011). Advances in mucoadhesion and mucoadhesive polymers. Macromol. Biosci..

