# Supplementary material for: An Alternative Device for the Topical Treatment of Oral Cancer: Development and Ex-Vivo Evaluation of Imiquimod-Loaded Polysaccharides Formulations
Source: Pharmaceutics. 2022 Nov 23;14(12):2573. doi: 10.3390/pharmaceutics14122573 (PMC9785792; doi:10.3390/pharmaceutics14122573)
Supplement: Supplementary file 1 [file pharmaceutics-14-02573-s001.zip › pharmaceutics-2045396-supplementary.pdf]

Supplemental material

Table S1. Percentage composition of the preliminary formulations.

Component	Gel 1	Gel ISO	Gel ISO2
IMQ	0.05	0.06	0.19
Alginate	0.95	0.43	0.43
CMChit	0.95	-	-
CaCl2	0.02	0.03	0.03
Propylene glycol	0.99	4.33	4.33
Water	97.05	93.10	92.98
Xanthan gum	-	0.87	0.87
Isostearic acid	-	1.17	1.17

Table S2. P values obtained from t-test analysis for IMQ amount retained and permeated across porcine skin for all the formulation prepared.

Formulation	Micelles	CMChit+A gel	A+X gel	X gel	A+X Film	A+X Film with backing	X Film no crosslinked	X Film no crosslinked with backing	X Film 120°C 20 min with backing
Micelles	-	**0.001**; **0.008**	0.175; **0.020**	0.796; 0.572	0.383; 0.226	**0.028**; **0.007**	0.130; **0.012**	0.063; 0.863	0.148; **0.001**
CMChit+A gel	**0.001**; **0.008**	-	0.087; **0.002**	0.052; **0.021**	0.115;0.088	**0.004**;0.085	0.085; 0.673	**0.047**; **0.008**	**0.001**; 0.171
A+X gel	0.175; **0.020**	0.087; **0.002**	-	0.305; 0.180	0.813; 0.018	0.955; **0.001**	0.918; **0.003**	0.056; **0.026**	**0.035**; **0.001**
X gel	0.796; 0.572	0.052; **0.021**	0.305; 0.180	-	0.433; 0.198	0.239; **0.045**	0.271; **0.021**	0.067; 0.639	0.537; **0.006**
A+X Film	0.383; 0.226	0.115;0.088	0.813; **0.018**	0.433; 0.198	-	0.743; 0.356	0.744; 0.076	0.058; 0.201	0.089; **0.017**
A+X Film with backing	**0.028**; **0.007**	**0.004**; 0.085	0.955; **0.001**	0.239; **0.045**	0.743; 0.356	-	0.934; 0.086	0.055; **0.007**	**0.011**; **0.003**
X Film no crosslinked	0.130; 0.012	0.085; 0.673	0.918; **0.003**	0.271; **0.021**	0.744; 0.076	0.934; 0.086	-	0.055; **0.011**	0.055; **0.011**
X Film no crosslinked with backing	0.063; 0.863	**0.047**; 0.008	0.056; **0.026**	0.067; 0.639	0.058; 0.201	0.055; **0.007**	0.055; **0.011**	-	**0.033**; **0.001**
X Film 120°C 20 min with backing	0.148; **0.001**	**0.001**; 0.171	**0.035**; **0.001**	0.537; **0.006**	0.089; **0.017**	**0.011**; **0.003**	**0.027**; 0.467	**0.033**; **0.001**	-
